# A Novel Association between Femoroacetabular Impingement and Anterior Knee Pain

**DOI:** 10.1155/2015/937431

**Published:** 2015-09-14

**Authors:** Vicente Sanchis-Alfonso, Marc Tey, Joan Carles Monllau

**Affiliations:** ^1^Hospital 9 de Octubre, Valle de la Ballestera 59, 46015 Valencia, Spain; ^2^Hospital del Mar, Passeig Marítim 25-29, 08003 Barcelona, Spain

## Abstract

*Background*. For a long time it has been accepted that the main problem in the anterior knee pain (AKP) patient is in the patella. Currently, literature supports the link between abnormal hip function and AKP. *Objective*. Our objective is to investigate if Cam femoroacetabular impingement (FAI) resolution is related to the outcome in pain and disability in patients with chronic AKP recalcitrant to conservative treatment associated with Cam FAI. *Material and Methods*. A retrospective study on 7 patients with chronic AKP associated with FAI type Cam was performed. Knee and hip pain were measured with the visual analogue scale (VAS), knee disability with the Kujala scale, and hip disability with the Nonarthritic Hip Score (NAHS). *Results*. The VAS knee pain score and VAS hip pain score had a significant improvement postoperatively. At final follow-up, there was significant improvement in all functional scores (Kujala score and NAHS). *Conclusion*. Our finding supports the link between Cam FAI and AKP in some young patients. Assessment of Cam FAI should be considered as a part of the physical examination of patients with AKP, mainly in cases with pain recalcitrant to conservative treatment.

## 1. Introduction

Anterior knee pain (AKP) (i.e., pain perceived in the anterior aspect of the knee following exclusion of other objective causes of pain) is one of the most frequent reasons for consultation within knee conditions in adolescents and young adults [1]. However, despite the high prevalence of this disorder, its etiology is not completely understood, which explains the sometimes unpredictable results of its treatment [1].

For a long time, it has been accepted that the main problem in the AKP patient is in the patella. However, currently, literature supports the link between abnormal hip function and AKP [2–7]. We have observed clinically a relationship between AKP and Cam femoroacetabular impingement (FAI). The objective of the present study is to investigate if Cam FAI resolution is related to the outcome in pain and disability in patients with chronic AKP recalcitrant to conservative treatment. To our knowledge, there are no previous studies in AKP patients that analyze the link between pain and Cam FAI.

## 2. Material and Methods

**Figure 1:**
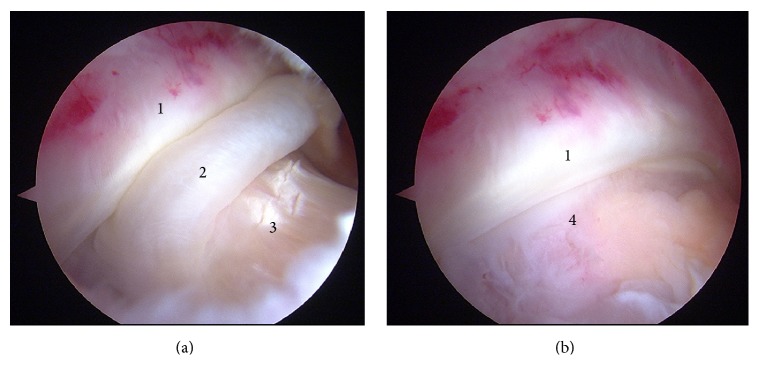
Anterior left knee pain and symptomatic left Cam femoroacetabular impingement. (a) 90° of hip flexion with maximum internal rotation. (b) The same image at 90° of hip flexion with maximum external rotation. (1) Labrum, (2) synovial tissue, (3) femoral neck, and (4) femoral head.

A computer-assisted search of our surgical database was performed to find patients who had AKP associated with Cam FAI. Seven consecutive patients (6 males, 1 female) were identified from May 2011 to April 2013. All of them were available for follow-up. All patients gave their informed consent to participate in the study. This study was approved by our Institutional Review Board. Patient characteristics are presented in Table 1. The mean age was 32.5 years ± 9.07 (range, 21 to 46 years). All patients underwent primary femoral neck osteoplasty. During arthroscopy we confirmed the impingement mechanism with the hip at 90° of flexion and maximum internal rotation (Figure 1(a)). With external femoral rotation we avoid the impingement and therefore the hip pain (Figure 1(b)). After hip surgery no specific physiotherapy treatment of the AKP was performed. All of the patients went to the orthopaedic surgeon for the first time due to AKP recalcitrant to conservative treatment, appearing as groin pain always later. The mean duration of the knee pain from the onset to the moment of hip surgery was 26.14 months ± 17.57 (range, 3 to 53 months). The mean length of follow-up for this cohort was 16.85 months ± 8.64 (range, 6 to 29 months).

Inclusion criteria included the following: (1) pain on the anterior aspect of the knee following exclusion of other objective causes of pain, (2) knee Visual Analogic Scale (VAS) of 5 or higher, (3) normal image studies of the knee, and (4) presence of disabling pain and disability which had not responded to a previous physical therapy treatment for at least 3 months. Normal image studies included torsional computed tomography or magnetic resonance image with femoral torsion of 5° to 20°, tibial torsion of 20° to 35°, TT-TG distance < 20 mm, patellar tilt < 20°, lateral radiograph with Caton Deschamps index of 0.8 to 1.2, and telemetric radiograph with mechanical axis of lower limb of 5° to −5° (varus to valgus) and limb length discrepancy less than 1.5 cm. Cam FAI was confirmed by groin pain, positive impingement test, positive decompression test, and radiograph with alpha angle at Dunn view greater than 50°.

Patients were studied with VAS for AKP and groin pain, Kujala knee score and Nonarthritic Hip Score (NAHS). Pain VAS was used to evaluate pain intensity preoperatively and at the time of final follow-up. VAS scales have been used in several studies to measure subjects' level of pain and it has demonstrated good reliability and concurrent validity when compared with other methods of pain measurement [8, 9]. The Kujala knee score has been widely used to evaluate disability in patellofemoral conditions [10]. The Kujala score was recorded preoperatively and at the time of final follow-up. The NAHS is a reproducible, short, self-administered hip score, with internal consistency, designed for use in young patients [11]. The NAHS was recorded preoperatively and at the time of final follow-up.

*Statistical Analysis*. Descriptive statistics for each of the measures evaluated was calculated. Quantitative variables were described with means and standard deviations. Mean preoperative and postoperative hip VAS, knee VAS, Kujala scale, and NAHS values were compared using the Wilcoxon test in the Statistical Package for Social Sciences (SPSS), version 16.0 (SPSS Inc., Chicago, Illinois, USA), with statistical significance for* p* values defined as *p* < 0.05.

## 3. Results

Detailed results are presented in Table 1. The VAS knee pain score had a significant improvement from 6.78 ± 1.46 preoperatively to 1.07 ± 1.30 postoperatively (*p* = 0.014). The VAS hip pain score had a significant improvement from 4.35 ± 2.13 preoperatively to 0.85 ± 0.89 postoperatively (*p* = 0.018). At final follow-up, there was significant improvement in all functional scores (Kujala score and NAHS) (*p* = 0.018). The Kujala score improved from 44.85 ± 11.99 preoperatively to 93 ± 2.51 postoperatively. The NAHS improved from 60.53 ± 15.37 preoperatively to 90.67 ± 3.74 postoperatively. There were no perioperative complications. The alpha angle decreased from 67.42 ± 4.57 preoperatively to 42.71 ± 6.29 postoperatively (*p* = 0.018).

## 4. Discussion

The most important finding of this study was the link between abnormal hip function, in our case Cam FAI, and AKP in young patients. In our experience, all of our patients went to the orthopedic surgeon for the first time due to AKP that was recalcitrant to a correct conservative treatment. The hip pain appeared always later.

A clear understanding of the cause of AKP is crucial to perform the treatment. Our findings are in agreement with a growing body of literature linking abnormal hip function with AKP [2–7]. Clinically, in patients with Cam FAI we have observed an external femoral rotation that we interpret as a defense mechanism to avoid the impingement and therefore the hip pain [1]. We believe that this functional femoral rotation could be responsible for patellofemoral imbalance and therefore pain [1]. On the same wavelength, some authors have analyzed the importance of external femoral rotation in the genesis of AKP [12–18]. Cibulka and Threlkeld-Watkins [3] reported an unusual case of patellofemoral pain in a patient with excessive hip external rotation. Karaman et al. [13] have shown that a femoral rotational malalignment greater than or equal to 10°, both external and internal, after closed intramedullary nailing of femoral shaft fractures, affected the patellofemoral joint provoking AKP while climbing stairs. Finally, Yildirim et al. [17] have observed that an external rotational deformity of the femur greater than 10° provokes AKP.

The major limitation of our study was the small number of included patients (*n* = 7). The great strength of our study is that it is the first one that analyzes the link between AKP pain and Cam FAI.

In spite of the limitations of this study, our findings could have meaningful potential implications for clinicians in the evaluation and treatment of AKP and for researchers in future studies on etiopathogenesis of AKP. The results of our study support the link between abnormal hip function, in our case Cam FAI, and AKP in young patients. Patellofemoral joint is very sensitive to this specific hip condition. That is, in some patients the underlying cause of AKP cannot be in the patellofemoral joint. From a clinical point of view, our data suggest that assessment of Cam FAI should be considered as a part of the physical examination of patients with AKP, mainly in cases with pain recalcitrant to conservative treatment. Cam FAI surgery is a safe and efficacious treatment [19]. Therefore, once a causative relationship between Cam FAI and AKP is established, we recommend Cam surgery. FAI resolution, that is, the normalization of alpha angle, is related to the resolution of disability, that is, the increment of the Kujala score, and the resolution of knee pain, that is, the decrease of VAS.

However, more studies on larger patient series would be required to draw definitive conclusion. In addition, long-term follow-up is mandatory to further confirm our results.

## Figures and Tables

**Table 1:** Patient characteristics and detailed results.

Case	Age (yrs)	Sex	Preoperative VAS		Postoperative VAS		Kujala score		NAH Score		Alfa Angle		Duration of knee pain (m)	Follow-up (m)
			Knee	Hip	Knee	Hip	Preoperative	Postoperative	Preoperative	Postoperative	Preoperative	Postoperative		
1	24	M	8,5	2,5	3,5	2,0	44	91	55,00	86,25	68	36	26	24
2	46	M	5,0	3,0	0,0	1,0	62	95	65,00	87,95	68	47	11	12
3	39	M	6,0	4,0	1,0	2,0	46	96	68,00	92,00	72	48	50	29
4	39	M	5,0	2,0	0,0	1,0	50	91	70,00	92,00	70	44	53	24
5	21	M	7,0	5,0	2,0	0,0	48	96	75,00	91,00	70	48	16	6
6	31	M	8,0	6,0	0,0	0,0	42	91	62,00	88,00	66	44	3	12
7	28	F	8,0	8,0	1,0	0,0	22	91	28,75	97,50	58	32	24	11
